# Multimorbidity and out-of-pocket expenditure on medicine in Europe: Longitudinal analysis of 13 European countries between 2013 and 2015

**DOI:** 10.3389/fpubh.2022.1053515

**Published:** 2023-01-05

**Authors:** Raffaele Palladino, Tianxin Pan, Stewart W. Mercer, Rifat Atun, Barbara McPake, Fabiana Rubba, Maria Triassi, John Tayu Lee

**Affiliations:** ^1^Department of Primary Care and Public Health, School of Public Health, Imperial College London, London, United Kingdom; ^2^Department of Public Health, University “Federico II” of Naples, Naples, Italy; ^3^Interdepartmental Research Center in Healthcare Management and Innovation in Healthcare (CIRMIS), University “Federico II” of Naples, Naples, Italy; ^4^Health Economics Unit, Center for Health Policy, Melbourne School of Population and Global Health, The University of Melbourne, Melbourne, VIC, Australia; ^5^Centre for Population Health Sciences, The Usher Institute, The University of Edinburgh, Edinburgh, United Kingdom; ^6^Department of Global Health and Population, Harvard T H Chan School of Public Health and Department of Global Health and Social Medicine, Harvard Medical School, Harvard University, Boston, MA, United States; ^7^Melbourne School of Population and Global Health, Nossal Institute for Global Health, The University of Melbourne, Melbourne, VIC, Australia

**Keywords:** multimorbidity, out-of-pocket, out-of-pocket (OOP) expenses, medicines, health system, universal health coverage

## Abstract

**Background:**

Many European Health Systems are implementing or increasing levels of cost-sharing for medicine in response to the growing constrains on public spending on health despite their negative impact on population health due to delay in seeking care.

**Objective:**

This study aims to examine the relationships between multimorbidity (two or more coexisting chronic diseases, CDs), complex multimorbidity (three or more CDs impacting at least three different body systems), and out-of-pocket expenditure (OOPE) for medicine across European nations.

**Methods:**

This study utilized data on participants aged 50 years and above from two recent waves of the Survey of Health, Aging, and Retirement in Europe conducted in 2013 (*n* = 55,806) and 2015 (*n* = 51,237). Pooled cross-sectional and longitudinal study designs were used, as well as a two-part model, to analyse the association between multimorbidity and OOPE for medicine.

**Results:**

The prevalence of multimorbidity was 50.4% in 2013 and 48.2% in 2015. Nearly half of those with multimorbidity had complex multimorbidity. Each additional CD was associated with a 34% greater likelihood of incurring any OOPE for medicine (Odds ratio = 1.34, 95% CI = 1.31**–**1.36). The average incremental OOPE for medicine was 26.4 euros for each additional CD (95% CI = 25.1**–**27·7), and 32.1 euros for each additional body system affected (95% CI 30.6**–**33.7). In stratified analyses for country-specific quartiles of household income the average incremental OOPE for medicine was not significantly different across groups.

**Conclusion:**

Between 2013 and 2015 in 13 European Health Systems increased prevalence of CDs was associated with greater likelihood of having OOPE on medication and an increase in the average amount spent when one occurred. Monitoring this indicator is important considering the negative association with treatment adherence and subsequent effects on health.

## Introduction

Multimorbidity, defined as the presence of two or more coexisting chronic diseases (CDs) (1), is on the rise globally, and its prevalence is expected to rise further as the population ages (1–6). Because of the high complexity of the care they require, people with multimorbidity incur greater health care expenditure and poorer health outcomes, with these relationships being substantially stronger when multimorbidity affects multiple body systems (7). Multimorbidity may have a dipropionate impact on the poor, as studies have shown that the prevalence of multimorbidity is higher among them in high-income nations, and they are more vulnerable to medical costs associated with multimorbidity (8).

While many nations throughout the world are making progress toward universal health coverage (UHC), recent research have indicated that financial protection for medical costs is being eroded in several European countries as a result of austerity measures and reduced public investment on health (2). According to recent studies on UHC, cost sharing policies have been implemented in Europe throughout the previous decade of public budget restraint, with most of the policy changes related to medicine and outpatient care (2, 9). Monitoring out-of-pocket expenditure (OOPE) on healthcare trends is crucial not just because of the financial burden associated with illness for individuals, but also because of its impact on patients' access to health care, medial adherence, and chronic disease management (2, 10–12).

Examining the influence of multimorbidity on OOPE for medicine is crucial for policy making because studies have indicated that medicine accounts for the majority of OOPE for people suffering from chronic conditions (13–15). According to a recent systematic study, an increase in the number of chronic conditions was linked with increased OOPE on medicines (13), with the elderly population being more susceptible to OOPE on medicine at all levels of multimorbidity (13, 16). Polypharmacy, which is compounded by the use of single disease-centered guidelines to manage persons with complex care needs, is common in people with multimorbidity and linked to an increase in OOPE on medicine (17). Although recent data reported that OOPE on medicine for the general population accounts for nearly or more than 20% of the health spending in many European countries, including Czech Republic, Estonia, Germany, Italy, Slovenia, and Spain (18), the majority of previous investigations were conducted in countries other than Europe (13), and none of these studies investigated the impact of OOPE on medicine by socioeconomic groups across European nations. To fill this important evidence vacuum, this study aims to investigate the relationship between multimorbidity and OOPE on medicine using longitudinal national representative data from 13 European Health Systems from 2013 to 2015, and whether this varied by respondents' socioeconomic position.

## Methods

### Data and sample

We used two waves of data from wave 5 (2013) and wave 6 (2015) of the Survey of Health, Aging, and Retirement in Europe (SHARE), a European panel database containing nationally representative samples of respondents aged fifty and over from 28 European countries and Israel (19). Respondents' sociodemographic factors, health status (including the presence of chronic illnesses and disability), and health care use and spending are all included in the data. SHARE's methodologies have been described in depth elsewhere (19). It is worth mentioning that while the SHARE dataset's fourth wave covered more nations, OOPE for medicine data was not collected in that wave. Also in wave 7, the bulk of respondents (80%) had missing information on their OOPE for medicine. We did not use wave 8 because it was disrupted by the COVID-19 pandemic outbreak. Data from the following 13 countries were considered: Austria, Belgium, Czech Republic, Denmark, Estonia, France, Germany, Italy, Luxembourg, Slovenia, Spain, Sweden, and Switzerland, which were preset in both waves 5 and 6. Residential care homes residents were excluded from our sample because they are expected to have different health seeking behavior than noninstitutionalized respondents.

In 2013 57,879 (wave 5) and in 2015 53,929 (wave 6) individuals aged 50 years and older did not live in a nursing facility. 55, 806 and 51,237 people, respectively, had comprehensive information on the variables of interest listed below. We employed an unbalanced sample of 65,206 individuals with 107,043 observations from the two waves.

### Variables

#### Multimorbidity

The main variable of interest was the number of coexisting CDs reported by each respondent. To assess multimorbidity, we considered 17 CDs, including 16 self-reported health conditions (heart attack/problem, hypertension, hypercholesterolemia, stroke/cerebral vascular illness, diabetes, cancer, peptic ulcer, stomach or duodenal ulcer, chronic lung disease, arthritis/rheumatism, Parkinson's disease, cataracts, hip or femoral fracture, other fracture, osteoarthritis, Alzheimer's disease/dementia/organic brain syndrome/senility/other significant cognitive impairment, other affective/emotional disorders), and one symptom-based health condition (depression). Participants were asked to answer the following question: “Has a doctor ever told you that you had/do you currently have any of the conditions listed on this card?” Clinical depression was the only chronic disease that was not defined based on the answer to this question. The EURO-D scale was used to measure and define it, in agreement with prior studies (1, 20), with scores of 4 or higher indicating the presence of clinically significant depressive symptoms. Asthma and kidney illness were not considered because they were not consistently asked about in the two waves.

Previous evidence suggested that individuals with complex multimorbidity, defined as the co-occurrence of three or more chronic diseases that affect at least three different body (organ) systems in one person (21, 22), have higher care needs, which might also translate into higher financial burden (17). Therefore, in our research we also assessed the presence of complex multimorbidity. Using the International Classification of Diseases, 10th revision (ICD-10), CDs were further divided into organ systems, with the following ten being included: neoplasms, endocrine, mental illness, nervous system, eye, circulatory system, respiratory system, digestive system, musculoskeletal or connective tissue, and fracture.

#### Outcome variables

OOPE on pharmaceuticals were the key outcome of interest. The enquiry “About how much did you pay altogether for drugs in the last 12 months? (Include both doctor-prescribed and non-prescription drugs)” led to OOPE on medicines. The OOPE on medicine is expressed in Euros and is adjusted for inflation to the year of the latest data collection (2017) to allow comparisons across time.

#### Covariates

Additional study variables included age (50–59, 60–69, or 70 and older), sex (male, female), marital status (married or in a civil partnership, others), residential country, educational attainment (less than upper secondary, upper secondary, or tertiary education), household income per capita (in quartiles within each country for each wave respectively; the poorest being Q1, the richest being Q4), as proxy of socio-economic position (SEP).

### Statistical analyses

We first assessed the prevalence of multimorbidity and complex multimorbidity in each nation, as well as by age group and socioeconomic position. In this analysis, sample weights in SHARE for cross-sectional data were used to ensure that our estimates were comparable throughout time. We further investigated patterns of multimorbidity and presented the percentage of people who had each illness dyad.

We used two-part model to assess the connections between multimorbidity and OOPE in medicine. When health expenditure represents the population as a whole, rather than just the users of health care, the distributions usually display substantial skewness and have a large mass point at zero (i.e., truncated at zero) (23–25). In our sample, nearly one-third of observations have zero expenditures on medicine. The health economics literature has settled on the two-part model as the best way to model a dependent variable with a large mass at zero and many positive values (25, 26). Therefore, we first modeled the probability that a person has any OOPE on medicine with a logit model using the full sample and then estimated a generalized linear model (GLM) on the subset of people who have any OOPE on medicine. Following literature (27), we used a Box- Cox test to determine which power function for transforming the dependent expenditure to be closet to symmetric; and the estimated coefficient was 0.06, corresponding to the natural log transformation. We used modified Park test to determine the distribution family; we observed an estimated coefficient of 1.58 which suggested the Gamma distribution. In summary, we use the log link and the gamma distribution for the GLM model. We presented estimated adjusted odds ratios (OR) and coefficients (with 95 percent confidence intervals) from first part and second part of the regression model, respectively. We further estimated average incremental expenditure (in euros) on medicine (combined marginal effects from both parts) of each additional CD from the model (25, 28).

Analyses were controlled for the covariates listed above. We used a pooled sample of all nations to run the model, which contained dummy variables for each country. To account for the fact that some people appeared in both waves, standard errors were clustered at the individual level to control for serial correlation. Sub-group analysis was carried out by repeating the analysis for each socioeconomic group and each country separately and we reported marginal effects of CDs on OOPE on medicine. STATA 14.0 was used for all statistical analyses.

Two sets of sensitivity analysis were performed. First, instead of using continuous variables to represent the number of CDs, we used a categorical variable to represent the number of conditions (0, 1, 2, 3, and 4 and more conditions) and repeated the primary analysis. Second, we used Cragg's hurdle model for our main analysis, which has also been used in literature to deal with health expenditure or outcomes with mass zeros (29, 30).

## Results

### Sample characteristics

We analyzed 107,043 observations from 65,206 different people. 54% of our sample were female. In 2013, 66.9 percent of respondents were 60 years old or older, compared with 66.4 percent in 2015. In 2015, 60.4 percent of respondents had completed at least secondary school, and 30.8 percent were employed (Table 1).

**Table 1:** Sample characteristics of respondents from 13 European countries.

	**2013**		**2015**	
	***N***	**%**	***N***	**%**
**Age group**				
50–59 years	14,854	33.1%	11,786	33.6%
60–69 years	19,749	29.5%	18,592	29.9%
70+ years	21,203	37.4%	20,859	36.5%
**Gender**				
Male	24,895	46.0%	22,469	46.3%
Female	30,911	54.0%	28,768	53.7%
**Marital status**				
Other	16,658	35.0%	15,806	35.5%
Married or in a civil partnership	39,148	65.0%	35,431	64.5%
**Educational attainment**				
Less than upper secondary	21,814	42.7%	19,277	39.6%
Upper secondary	21,482	37.1%	20,062	38.9%
Above	12509	20.2%	11,898	21.5%
**Household income**				
Q1	13,971	26.2%	12,826	25.7%
Q2	13,936	24.7%	12,795	24.3%
Q3	13,953	24.6%	12,809	24.4%
Q4	13,946	24.6%	12,806	25.5%
**Country**				
Austria	3,965	2.5%	3,068	2.6%
Germany	5,433	28.1%	4,214	28.5%
Sweden	4,376	3.0%	3,740	3.0%
Spain	6,139	13.6%	4,953	13.7%
Italy	4,498	20.3%	4,883	20.1%
France	4,307	20.4%	3,673	20.1%
Denmark	3,926	1.7%	3,554	1.7%
Switzerland	2,932	2.5%	2,694	2.5%
Belgium	5,312	3.4%	5,412	3.4%
Czech Republic	5,220	3.2%	4,516	3.2%
Luxembourg	1,509	0.1%	1,483	0.1%
Slovenia	2,829	0.7%	3,972	0.7%
Estonia	5,360	0.4%	5,075	0.4%
N	55,806		51,237

Descriptive statistics were calculated using the survey weights provided.

### Prevalence of multimorbidity and complex multimorbidity

**Figure 1:**
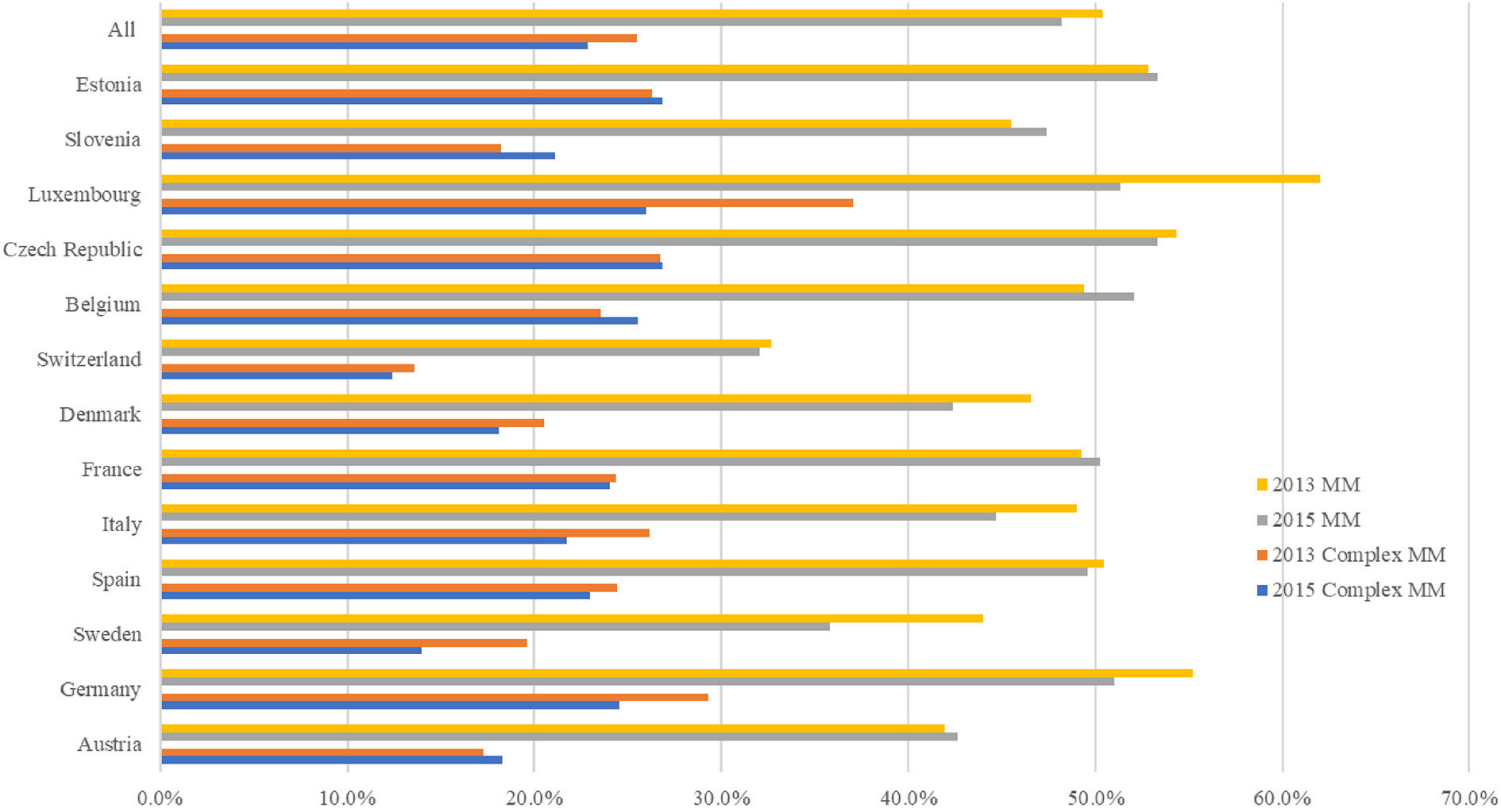
Prevalence of multimorbidity and complex multimorbidity among people ages 50 and older in 13 European countries, 2013–2015. Complex sample weight applied to the analysis. MM, multimorbidity, defined as the presence of two or more chronic diseases; Complex MM, complex multimorbidity, defined as having three or more chronic diseases impacting at least three different body systems in one person.

Figure 1 shows the prevalence of multimorbidity and complex multimorbidity in each country in 2013 and 2015. In 2013 and 2015, the prevalence of multimorbidity was 50.4 percent and 48.2 percent, respectively, and the prevalence of complex multimorbidity was 25.5 percent and 22.9 percent. Nearly half of individuals (49.0 percent) with multimorbidity had complex multimorbidity. In 2015, the prevalence of multimorbidity ranged from 32.1 percent (Switzerland) to 53.3 percent (Estonia) and ranged from 12.4 percent (Switzerland) to 26.9 percent (Estonia) for complex multimorbidity. Though there was a decrease in the prevalence of multimorbidity and complex multimorbidity within our full sample, five countries registered an increase (Austria, Belgium, France, Slovenia and Estonia) from 2013 to 2015. Supplementary Table S1 reports the prevalence of multimorbidity and complex multimorbidity in each country.

**Figure 2:**
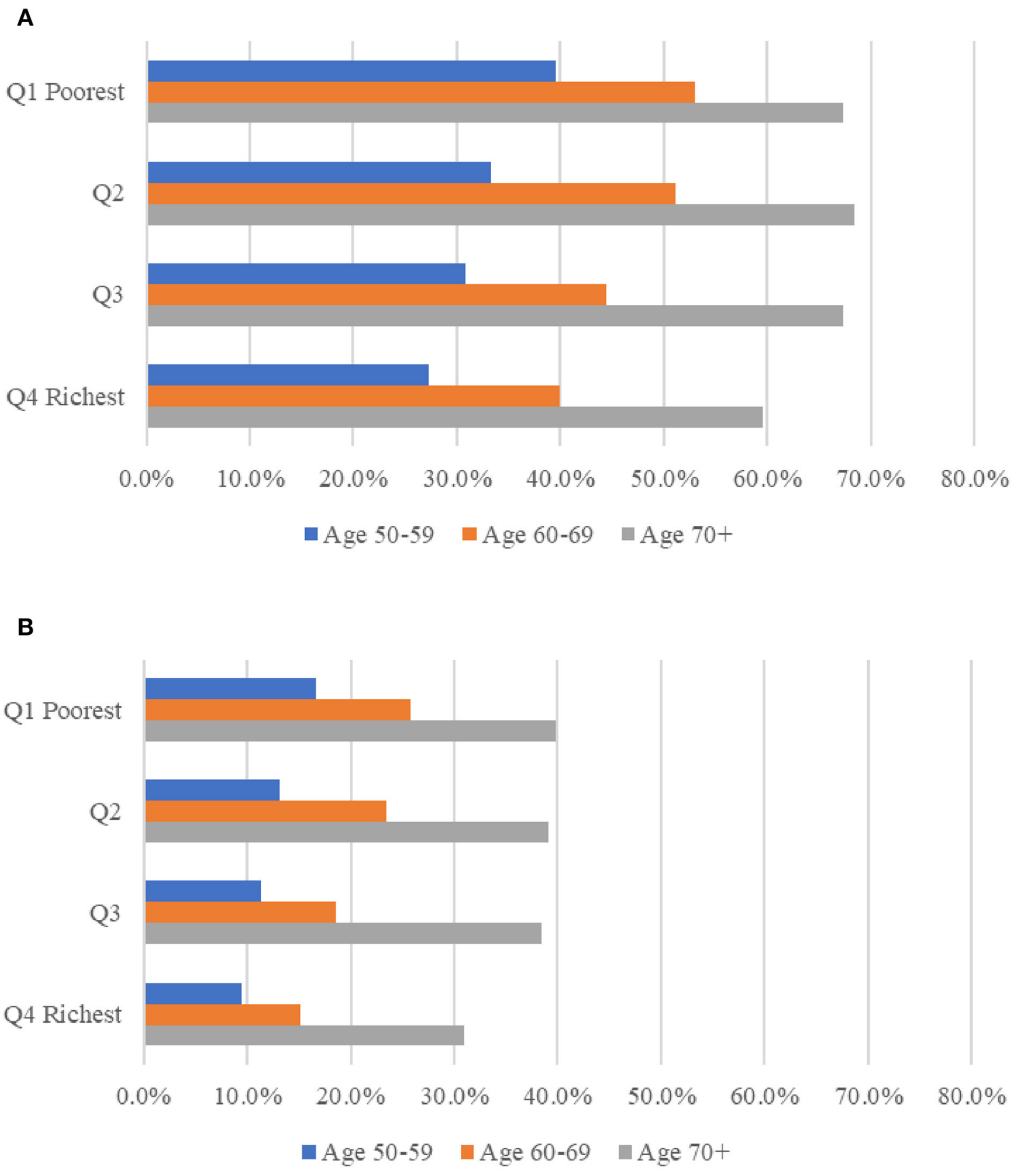
Prevalence of multimorbidity **(A)** and complex multimorbidity **(B)** among people ages 50 and older in 13 European countries, analysis in pooled sample of 2013 and 2015, by age and socio-economic position. Complex sample weights applied to the analyses. MM, multimorbidity, defined as the presence of two or more of chronic diseases; Complex MM, complex multimorbidity, defined as having three or more chronic diseases impacting at least three different body systems in one person. Socio-economic position measured using household income per capita, in quartiles within each country for each wave respectively; the poorest being Q1 = 1, the richest being Q4 = 4.

Multimorbidity is depicted in Figure 2 by age group and socioeconomic position. Multimorbidity and complex multimorbidity were shown to be more common as people became older. The prevalence was higher among respondents from lower socioeconomic groups within their country. Except for the richest group, the prevalence of multimorbidity was comparable across other population groups (between 67.3 percent and 68.5 percent) among respondents aged 70 and older. The prevalence figures are presented in Supplementary Table S2.

**Figure 3:**
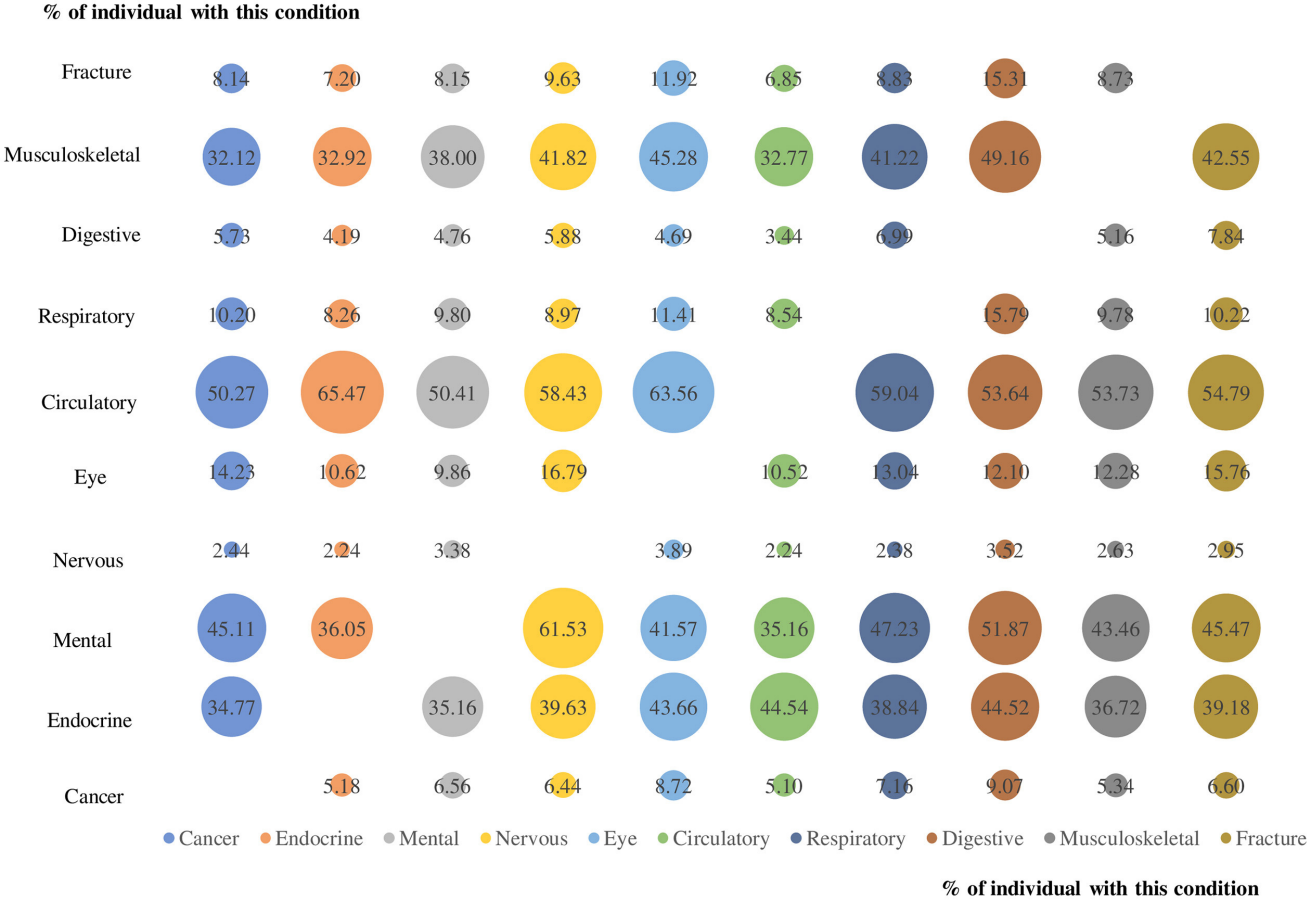
The prevalence of co-existing chronic diseases from different body systems for each body system condition for people with multimorbidity. Numbers in the bubble and bubble size represents the prevalence of co-existing chronic diseases from the two corresponding body systems.

On average, multimorbidity affected 1.7 body systems (95%CI 1.67–1.69) in 2013 and 1.6 body systems (95%CI 1.55–1.57) in 2015 (Supplementary Table S3), with circulatory system being the most affected body system in both years (44.4%, 95%CI 44.1–44.8% in 2013 and 43.1%, 95%CI 42.8–43.5% in 2015). Figure 3 depicts the prevalence of co-existing CDs from various body systems in people with multimorbidity. The most common dyad was circulatory system condition and endocrine condition (65.5 percent), followed by circulatory and eye condition (63.6 percent) and mental illness and nervous system condition (61.5 percent).

### Associations between multimorbidity and out-of-pocket expenditure on medicine

On average, among those who occurred relevant costs, total OOPE increased from 331 to 338 euros from 2013 to 215, with more than 50% of the total OOPE spent on medicine (50.2% in 2013 and 52.8% in 2015). The country with the largest proportion of total OOPE spent on medicine in 2015 was Estonia (71.1%), while the lowest was Switzerland (16.5%; Supplementary Table S4).

Table 2 displays the results of a two-part model that combines logit regression with GLM. According to the logit, each additional CD was associated with a 34% greater likelihood of incurring OOPE on medicine (OR = 1.34, 95% CI = 1.31–1.36). The GLM model suggests that each additional CD was associated with an increase in OOPE spending (regression coefficient 0.15, 95% CI = 0.14–0.16). We found that the average incremental spending of each additional CD was 26.4 euros (95%CI 25.1–27.7), according to the mean marginal effect incorporating both portions of the two-part model.

**Table 2:** Association between multimorbidity with OOPE on medicine among people ages 50 and older in 13 European countries between 2013 and 2015.

	**First part Logit**		**Second part GLM**		**Overall**	
	**Coefficient**	**95% CI**	**Coefficient**	**95% CI**	**Margins**	**95% CI**
Number of CDs	1.34^***^	(1.31–1.36)	0.15^***^	(0.14–0.16)	26.39^***^	(25.10–27.68)
**Age groups (ref: 50–59 years)**						
60–69	1.00	(0.94–1.08)	0.07^***^	(0.02–0.12)	7.99^***^	(2.50–13.49)
70+	0.97	(0.90–1.04)	0.18^***^	(0.13–0.22)	19.90^***^	(14.40–25.41)
**Gender (ref: male)**						
Female	1.26^***^	(1.19–1.33)	0.05^**^	(0.01–0.08)	12.06^***^	(7.46–16.65)
**Marital status (ref: other)**						
Married	1.06^*^	(1.00–1.13)	0.07^***^	(0.03–0.11)	9.62^***^	(4.92–14.32)
**Educational attainment (ref: less than secondary school)**						
Upper secondary	1.28^***^	(1.19–1.38)	0.02	(−0.03–0.06)	9.01^***^	(3.34–14.69)
Tertiary	1.30^***^	(1.19–1.42)	0.10^***^	(0.04–0.16)	19.67^***^	(12.35–26.99)
**Socio-economics position (ref: Q1 poorest)**						
Q2	1.28^***^	(1.20–1.37)	−0.04^**^	(−0.08–−0.00)	2.08	(−3.05–7.22)
Q3	1.42^***^	(1.32–1.53)	−0.03	(−0.07–0.02)	6.89^**^	(1.11–12.68)
Q4	1.28^***^	(1.19–1.39)	0.05^*^	(0.00–0.09)	12.96^***^	(6.69–19.23)
**Country (ref: Austria)**						
Germany	1.70^***^	(1.55–1.86)	−0.61^***^	(−0.66–−0.56)	−50.13^***^	(−57.33–−42.92)
Sweden	3.01^***^	(2.71–3.35)	−0.43^***^	(−0.48–−0.38)	−19.26^***^	(−26.63–−11.89)
Spain	1.31^***^	(1.16–1.49)	−0.84^***^	(−0.92–−0.76)	−72.82^***^	(−81.02–−64.62)
Italy	1.25^***^	(1.14–1.38)	−0.05^*^	(−0.10–0.01)	3.48	(−5.27–12.22)
France	0.68^***^	(0.62–0.74)	−0.83^***^	(−0.90–−0.76)	−86.26^***^	(−93.49–−79.03)
Denmark	2.52^***^	(2.29–2.77)	−0.08^***^	(−0.13–−0.02)	25.11^***^	(16.22–34.00)
Switzerland	0.63^***^	(0.57–0.69)	0.27^***^	(0.20–0.33)	12.38^**^	(1.50–23.26)
Belgium	3.78^***^	(3.40–4.19)	0.19^***^	(0.14–0.24)	89.91^***^	(79.88–99.94)
Czech Republic	3.62^***^	(3.14–4.18)	−0.97^***^	(−1.03–−0.91)	−66.90^***^	(−74.15–−59.65)
Luxembourg	1.67^***^	(1.47–1.90)	0.12^***^	(0.04–0.19)	42.99^***^	(28.76–57.23)
Slovenia	0.51^***^	(0.47–0.56)	−0.98^***^	(−1.06–−0.89)	−98.54^***^	(−105.73–−91.35)
Estonia	4.26^***^	(3.87–4.69)	−0.16^***^	(−0.21–−0.12)	24.27^***^	(16.71–31.83)
**Year**						
2015	1.05^**^	(1.00–1.10)	0.00	(−0.03–0.03)	1.78	(−1.99–5.56)

Estimates obtained from two-part model that the first part is modeled through a logit model to estimate the likelihood of incurring OOPE on medicine, and second part using a generalized linear model with gamma distribution and log link function to model the amount of OOPE on medicine if occurred. Standard errors were clustered at the individual level to control for serial correlation. Margins shows combined marginal effects from both parts of the two-part model. Confidence interval in parentheses.

^***^Statistical significance at the 1% level; ^**^statistical significance at the 5% level; ^*^statistical significance at the 10% level.

GLM, generalized linear model; CD, chronic diseases; CI, confidence interval.

The association between the number of CDs affecting various body systems and OOPE on medicine is shown in Table 3. The effects were similar to those considering CDs but were greater in their magnitude. The average extra expenditure of an additional number of body system was 32.1 euros (95 %CI = 30.6–33.7), according to the mean marginal effect.

**Table 3:** Association between complex multimorbidity with OOPE on medicine among people ages 50 and older in 13 European countries between 2013 and 2015.

	**First part Logit**		**Second part GLM**		**Overall**	
	**Coefficient**	**95% CI**	**Coefficient**	**95% CI**	**Margins**	**95% CI**
Number of CDs from different body systems	1.43^***^	(1.39–1.46)	0.18^***^	(0.17–0.20)	32.14^***^	(30.55–33.74)
**Age groups (ref: 50–59 years)**						
60–69	1.00	(0.93–1.07)	0.07^***^	(0.02–0.11)	7.48^***^	(1.96–12.99)
70+	0.95	(0.88–1.02)	0.17^***^	(0.13–0.22)	18.94^***^	(13.43–24.46)
**Gender (ref: male)**						
Female	1.25^***^	(1.18–1.32)	0.04^**^	(0.01–0.08)	11.38^***^	(6.77–15.99)
**Marital status (ref: other)**						
Married	1.06^*^	(1.00–1.13)	0.07^***^	(0.03–0.11)	9.71^***^	(5.01–14.41)
**Educational attainment (ref: less than secondary school)**						
Upper secondary	1.28^***^	(1.19–1.38)	0.02	(−0.03–0.06)	9.11^***^	(3.42–14.79)
Tertiary	1.30^***^	(1.20–1.42)	0.10^***^	(0.04–0.15)	19.78^***^	(12.49–27.06)
**Socio–economics position (ref: Q1 poorest)**						
Q2	1.28^***^	(1.20–1.37)	−0.05^**^	(−0.09–−0.01)	1.60	(−3.58–6.78)
Q3	1.42^***^	(1.32–1.53)	−0.03	(−0.08–0.02)	6.32^**^	(0.49–12.15)
Q4	1.28^***^	(1.18–1.38)	0.04	(−0.01–0.08)	11.69^***^	(5.39–17.98)
**Country (ref: Austria)**						
Germany	1.68^***^	(1.54–1.84)	−0.62^***^	(−0.67–−0.56)	−51.41^***^	(−58.67–−44.15)
Sweden	3.00^***^	(2.70–3.33)	−0.44^***^	(−0.49–−0.39)	−20.92^***^	(−28.33–−13.51)
Spain	1.32^***^	(1.16–1.49)	−0.84^***^	(−0.92–−0.76)	−73.54^***^	(−81.82–−65.25)
Italy	1.24^***^	(1.13–1.36)	−0.06^**^	(−0.11–−0.00)	1.62	(−7.17–10.41)
France	0.66^***^	(0.61–0.72)	−0.85^***^	(−0.92–−0.78)	−88.53^***^	(−95.78–−81.28)
Denmark	2.49^***^	(2.27–2.74)	−0.09^***^	(−0.14–−0.03)	23.64^***^	(14.67–32.61)
Switzerland	0.62^***^	(0.57–0.69)	0.26^***^	(0.19–0.32)	10.54^*^	(−0.36–21.45)
Belgium	3.73^***^	(3.36–4.14)	0.18^***^	(0.13–0.23)	88.06^***^	(77.95–98.17)
Czech Republic	3.60^***^	(3.12–4.15)	−0.97^***^	(−1.03–−0.91)	−67.70^***^	(−75.07–−60.33)
Luxembourg	1.63^***^	(1.44–1.86)	0.11^***^	(0.03–0.18)	40.68^***^	(26.47–54.89)
Slovenia	0.51^***^	(0.47–0.56)	−0.99^***^	(−1.07–−0.90)	−100.07^***^	(−107.27–−92.87)
Estonia	4.25^***^	(3.87–4.68)	−0.16^***^	(−0.21–−0.12)	24.76^***^	(17.12–32.41)
**Year**						
2015	1.05^**^	(1.01–1.10)	0.00	(−0.03–0.03)	1.83	(−1.96–5.61)

Estimates obtained from two-part model that the first part is modeled through a logit model to estimate the likelihood of incurring OOPE on medicine, and second part using a generalized linear model with gamma distribution and log link function to model the amount of OOPE on medicine if occurred. Standard errors were clustered at the individual level to control for serial correlation. Margins show combined marginal effects from both parts of the two-part model. Confidence interval in parentheses.

^**^Statistical significance at the 1% level; ^**^statistical significance at the 5% level; ^*^statistical significance at the 10% level.

GLM, generalized linear model; CD, chronic diseases; CI: confidence interval.

We repeated our main analysis using a categorical variable to represent the number of CDs (0, 1, 2, 3, and 4 and more conditions); the results were very comparable to those in the main analyses; and the results reveal that, when compared to persons without CD, those with four or more conditions spent additional 140.7 euros on medicine (Supplementary Tables S5, S6). We also used Cragg's hurdle model instead of two-part model for our main analysis. The marginal effects from hurdle model that combining the selection model and outcome model (25.9 euros for additional one CD on OOPE on medicine) were similar compared to those of two-part model (Supplementary Tables S7, S8).

### Associations between multimorbidity and out-of-pocket expenditure on medicine by SEP groups and country

**Figure 4:**
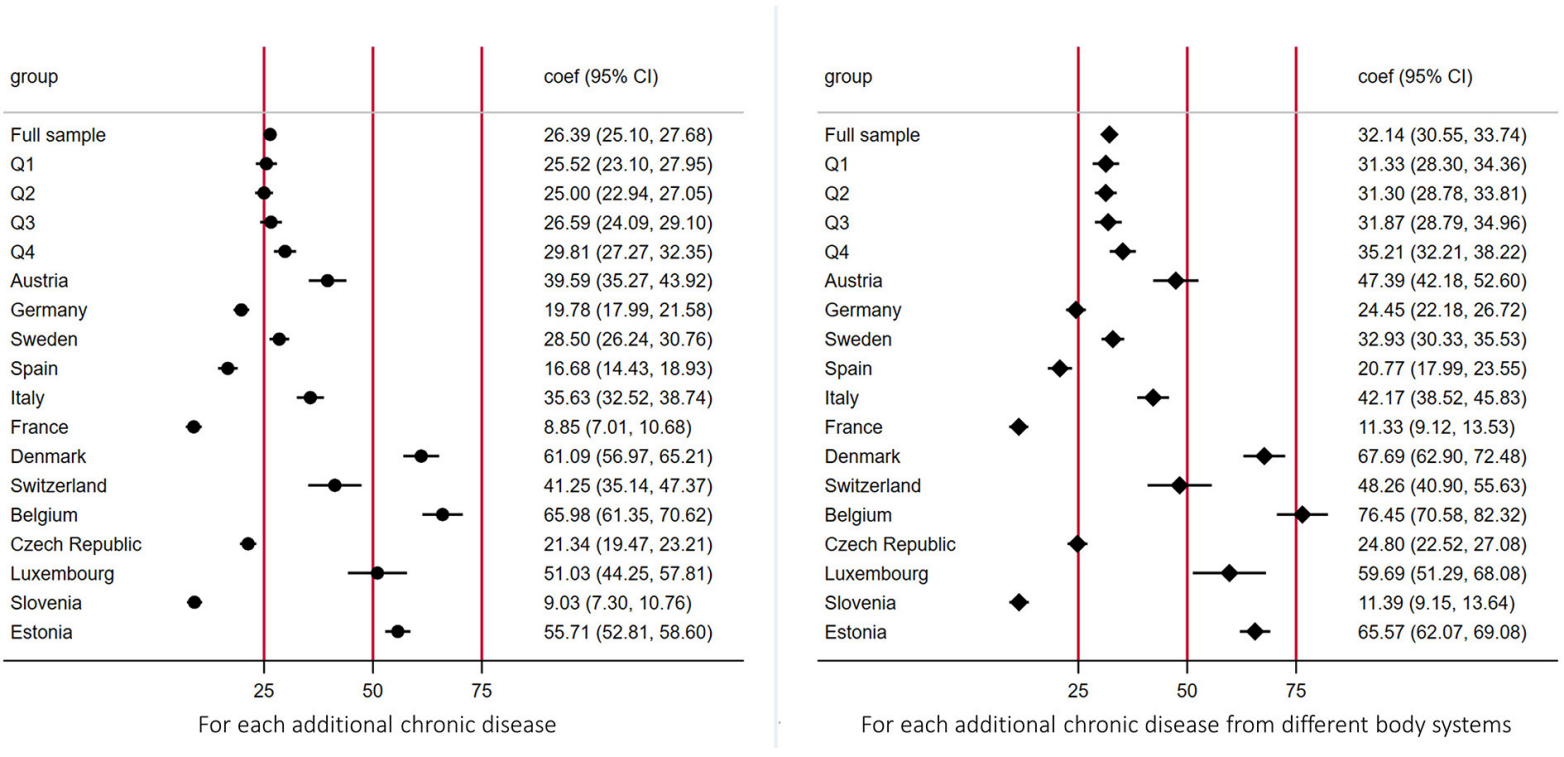
Average incremental out-of-pocket expenditures on medicine for each additional chronic diseases by economic position and country. The figure shows the average incremental out-of-pocket expenditures on medicine (i.e., marginal effects) for each additional chronic disease by economic status and country. Estimates were derived from a log link GLM model with gamma distribution. Socio-economic position measured using household income per capita, in quartiles within each country for each wave respectively; the poorest being Q1 = 1, the richest being Q4 = 4.

Figure 4 shows the average incremental OOPE on medicine (i.e., marginal effects) for an additional CD by economic status and country. People from the richest quartile of their country spent more OOPE for medicine for each additional CD, as compared with those from the poorest quartile (Q1: 25.52 euros, 95%CI = 23.10–27.95; Q2: 25.00, 95%CI = 22.94–27.05; Q3: 26.59, 95%CI = 24.09–29.10; Q4: 29.81, 95%CI = 27.27–32.35), although confidence intervals overlapped. Belgium, Denmark, and Estonia were the countries where people spent more on OOPE on average (66.0 euros, 61.0 euros and 55.7 euros respectively) for each additional number of CDs, while people from France and Slovenia spent much less on medicine out-of-pocket from CDs (8.9 and 9.0 euros). Trends were also confirmed when considering increase in CD from different body systems (Figure 4).

## Discussion

Our study is the first study to focus on the relationships between multimorbidity, complex multimorbidity, and OOPE for medicine across 13 European countries. We discovered that nearly half (48.2 percent) of adults aged 50 and older had multimorbidity in 2015, and 22.9 percent had complex multimorbidity. Although prevalence decreased slightly from 2013 levels (50.4 percent and 25.5 percent, respectively), patterns varied significantly between nations, which might partially be explained by the different level of integrated care model implementation for individuals with complex care needs in each country (1). The prevalence was highest among those over the age of 70 and those with a poor socioeconomic position. An increased number of CD was associated with an increased risk of experiencing OOPE on medicine and an increase in the average amount spent when one occurred. The average incremental expenditure of each additional CD was 26.4 euros. Whilst the marginal estimated expenditure of OOPE on medicine varied considerably across countries, no significant differences were observed in stratified analyses by country-specific quartiles of household income. When complex multimorbidity was included, the association between multimorbidity and OOPE on medicine was considerably stronger.

Consistent with previous research demonstrating the health system impoverishment exacerbating the burden of CD in European countries (1, 2, 5), our study discovered that multimorbidity is linked with a substantial rise in OOPE for medication across all socioeconomic categories. However, most of the previous studies focused on the effect of specific chronic conditions, whilst our study examined the impact of multimorbidity, particularly complex multimorbidity.

### Strength and limitations

To our knowledge the current study was the first to examine the impact of multimorbidity, particularly complex multimorbidity on OOPE on medicine in older adults in Europe. Additionally, our research was the first to use a panel data study methodology and to use nationally representative data from 13 European nations. Several limitations merit discussion. To begin, self-reported measures of CD and health care usage may have underestimated their frequency, especially among older adults and those with lower socioeconomic and educational status, who are more prone to underreport these variables (1, 31). Second, some of the country-specific differences we observed might be explained by differences in the Health Systems, especially in regard to cost-sharing policies. However, we conducted country-specific analyses for this specific reason and additionally controlled pooled analyses for countries as fixed effects to remove this variability. Third, the SHARE questionnaire does not contain questions regarding all CDs that are often included in clinical database research (32). Additional research investigating the impact of multimorbidity associated with other prevalent CDs (e.g., Alzheimer's disease, and mental health problems) and chronic infectious diseases (e.g., TB, AIDS, long coronavirus disease) is also needed. The social patterning of multimorbidity in Europe needs further study that should cover a broader variety of morbidities and more rigors measurements of both mental and physical health than has been previously documented. Future research with an appropriate powering will be necessary to determine the effect of multimorbidity as well as what specific multimorbidity dyads and complex multimorbidity contribute the most to the increasing of OOPE in general and specifically of OOPE on medicine.

### Policy implications

European health systems have lagged behind in responding to the growing burden of multimorbidity (1, 2, 5). Over the last few years, national and international guidelines have been produced to improve care for persons with multimorbidity (33, 34) and integrated care models targeting individuals with specific combinations of chronic diseases have been introduced in a number of countries. However, in the majority of European nations, the quality of care for persons with multiple morbidities remains suboptimal due to fragmented care pathways focused on a single condition, increasing the risk of polypharmacy and associated health expenditure (17, 35). Furthermore, the COVID-19 pandemic has exacerbated health system challenges by reducing integrated care pathways and geriatric rehabilitation services despite increased demand (36).

Multimorbidity is related with a higher reliance on healthcare and, thus, an increased expenditure on healthcare (1, 2). While new research indicates a global decline in OOPE, this is not the case for OOPE as a share of income (37). Additionally, our findings indicated that OOPE on medications is increasing in European countries over time. As medicine accounts for the majority of out-of-pocket expenditure (13–15), monitoring this statistic is critical, considering increased OOPE on medicine is connected with a larger chance of non-adherence, which has a negative effect on health (11).

While the increase in OOPE on medicine can be interpreted as further evidence of the erosion of the UHC in European Health Systems, we also discovered that those with lower socioeconomic position were less likely to incur OOPE on medicine. Whilst these findings might be implying that some form of social protection for cost-sharing policies remains in place, we also found that the average expenditure for each additional CD, estimated using marginal effects, was characterized by a positive but non statistically significant gradient when moving from the poorest to the richest group. These differences might be explained by several factors, which might impact the association between CD and OOPE on medicine differently. First, the efficacy of social protection policies might be limited without fully exempting those who are worse off from payment. Second, the most disadvantaged groups might be unable to afford to pay for the medications they need, which might result in delay in seeking care with negative impact on their health. These aspects warrant further research.

Ultimately, our findings indicating a significant increase in OOPE for medicine for individuals with multimorbidity are troubling. As CDs can last a lifetime, they can impose significant financial strain over time. Our findings emphasize the importance of enhancing financial protection for individuals with many comorbidities, as they will face a much-increased level of OOPE for medications.

## Conclusion

Although majority of the European Health Systems have yet to implement specific clinical guidelines, the management of multimorbidity should be an absolute public health priority, considering that over half of adults aged 50 and older in Europe have multimorbidity, and almost a quarter has complex multimorbidity. We found that increased number of CDs was associated with an increased risk of experiencing OOPE on medication and an increase in the average amount spent when one occurred. When complex multimorbidity was included, the association between multimorbidity and OOPE on medicine was considerably stronger. The average incremental OOPE on medicine associated with number of CDs varied substantially across countries but not between SEP groups. As medicine accounts for the majority of OOPE, monitoring this indicator is critical as it can be considered as a proxy of erosion of the UHC in European Health Systems and because increased OOPE on medicine is connected with a larger chance of non-adherence to treatment, which has a negative effect on health.

## Data availability statement

Publicly available datasets were analyzed in this study. Survey of Health, Aging and Retirement in Europe (SHARE) data are accessible after registration with the SHARE project at the following addresses: wave 5 (doi: 10.6103/SHARE.w5.710) and wave 6 (doi: 10.6103/SHARE.w6.710).

## Ethics statement

The studies involving human participants were reviewed and approved by Ethics Council of the Max Planck Society. The patients/participants provided their written informed consent to participate in this study.

## Author contributions

RP, TP, and JL conceived the study and devised the study methodology. RP and TP did the formal data analysis. RP, TP, SM, and JL wrote the first draft of the manuscript. RP and JL supervised the study. RP had final responsibility for the decision to submit for publication. All authors reviewed and edited the manuscript. All authors contributed to the article and approved the submitted version.
