# Supplementary material for: Multimorbidity and out-of-pocket expenditure on medicine in Europe: Longitudinal analysis of 13 European countries between 2013 and 2015
Source: Front Public Health. 2023 Jan 5;10:1053515. doi: 10.3389/fpubh.2022.1053515 (PMC9850796; doi:10.3389/fpubh.2022.1053515)
Supplement: Supplementary file 1 [file Data_Sheet_1.docx]

**Supplementary materials**

| **Table S1 Prevalence of multimorbidity among people ages 50 and older in 13 European countries in 2013 and 2015** | | | | | | | | | | | | |
| --- | --- | --- | --- | --- | --- | --- | --- | --- | --- | --- | --- | --- |
|  | Prevalence of multimorbidity | | | | | | Prevalence of complex multimorbidity | | | | | |
|  | 2013 | | | 2015 | | | 2013 | | | 2015 | | |
|  | % | 95% CI | | % | 95% CI | | % | 95% CI | | % | 95% CI | |
| Austria | 42·0% | 41·2% | 42·8% | 42·6% | 41·6% | 43·6% | 17·3% | 16·7% | 17·9% | 18·3% | 17·6% | 19·0% |
| Germany | 55·2% | 54·6% | 55·9% | 51·0% | 50·3% | 51·8% | 29·3% | 28·7% | 29·9% | 24·5% | 23·9% | 25·2% |
| Sweden | 44·0% | 43·2% | 44·7% | 35·8% | 34·9% | 36·6% | 19·6% | 19·0% | 20·2% | 14·0% | 13·4% | 14·6% |
| Spain | 50·4% | 49·3% | 51·6% | 49·6% | 48·2% | 50·9% | 24·4% | 23·6% | 25·3% | 23·0% | 22·1% | 24·0% |
| Italy | 49·0% | 48·2% | 49·8% | 44·7% | 43·9% | 45·5% | 26·1% | 25·4% | 26·8% | 21·7% | 21·1% | 22·4% |
| France | 49·2% | 48·5% | 50·0% | 50·2% | 49·4% | 51·1% | 24·3% | 23·7% | 25·0% | 24·0% | 23·4% | 24·7% |
| Denmark | 46·6% | 45·9% | 47·3% | 42·4% | 41·6% | 43·1% | 20·6% | 20·0% | 21·2% | 18·1% | 17·5% | 18·7% |
| Switzerland | 32·7% | 31·9% | 33·5% | 32·1% | 31·2% | 32·9% | 13·6% | 13·0% | 14·2% | 12·4% | 11·8% | 13·0% |
| Belgium | 49·4% | 48·6% | 50·1% | 52·1% | 51·3% | 52·9% | 23·6% | 23·0% | 24·2% | 25·6% | 24·9% | 26·2% |
| Czech Republic | 54·3% | 53·1% | 55·5% | 53·3% | 52·0% | 54·7% | 26·7% | 25·8% | 27·7% | 26·8% | 25·7% | 28·0% |
| Luxembourg | 62·0% | 60·8% | 63·2% | 51·3% | 49·9% | 52·7% | 37·1% | 35·9% | 38·3% | 26·0% | 24·8% | 27·2% |
| Slovenia | 45·5% | 44·5% | 46·5% | 47·4% | 46·6% | 48·3% | 18·2% | 17·5% | 19·0% | 21·1% | 20·4% | 21·8% |
| Estonia | 52·9% | 52·2% | 53·5% | 53·3% | 52·6% | 54·0% | 26·3% | 25·7% | 26·9% | 26·9% | 26·3% | 27·5% |
| All | 50·4% | 50·1% | 50·7% | 48·2% | 47·8% | 48·6% | 25·5% | 25·2% | 25·8% | 22·9% | 22·6% | 23·2% |

**Table S2 Prevalence of multimorbidity among people ages 50 and older by age group and socio-economic position in Europe**

|  | | | |
| --- | --- | --- | --- |
|  | Age 50-59 | Age 60-69 | Age 70+ |
| Prevalence of multimorbidity | | |  |
| Q4 Richest | 27·3% | 40·0% | 59·5% |
| Q3 | 30·8% | 44·4% | 67·4% |
| Q2 | 33·2% | 51·1% | 68·5% |
| Q1 Poorest | 39·5% | 53·1% | 67·3% |
| Prevalence of complex multimorbidity | | |  |
| Q4 Richest | 9·4% | 15·2% | 30·9% |
| Q3 | 11·3% | 18·5% | 38·4% |
| Q2 | 13·2% | 23·5% | 39·1% |
| Q1 Poorest | 16·7% | 25·7% | 39·8% |

| **Table S3 Number of chronic diseases and prevalence of conditions from different systems among the full sample in 2013 and 2015** | | | | |
| --- | --- | --- | --- | --- |
|  | **2013** | | **2015** | |
| **Average number of CDs** | 1.90 | (1.89 - 1.91) | 1.77 | (1.76 - 1.78) |
| **Average number of affected body systems** | 1.68 | (1.67 - 1.69) | 1.56 | (1.55 - 1.57) |
| **Prevalence** |  |  |  |  |
| Cancer | 5.9% | (5.8% - 6.1%) | 4.4% | (4.2% - 4.5%) |
| Endocrine system | 30.4% | (30.1% - 30.7%) | 29.3% | (29% - 29.7%) |
| Mental illness | 31.2% | (30.9% - 31.5%) | 30.1% | (29.7% - 30.4%) |
| Nervous system | 1.8% | (1.8% - 1.9%) | 1.7% | (1.6% - 1.7%) |
| Eye | 8.7% | (8.5% - 8.9%) | 7.1% | (7% - 7.3%) |
| Circulatory system | 44.4% | (44.1% - 44.8%) | 43.1% | (42.8% - 43.5%) |
| Respiratory system | 6.5% | (6.3% - 6.6%) | 6.2% | (6.1% - 6.4%) |
| Digestive system | 3.6% | (3.5% - 3.7%) | 2.8% | (2.6% - 2.9%) |
| Musculoskeletal system | 27.2% | (26.9% - 27.5%) | 26.3% | (26% - 26.6%) |
| Fracture | 8.0% | (7.8% - 8.2%) | 5.4% | (5.2% - 5.6%) |
| Note: Descriptive statistics were calculated using the survey weights provided. CD: chronic diseases. | | | | |

| **Table S4 mean out-of-pocket expenditures on inpatient and outpatient care and on medicine** | | | | | | | | | |
| --- | --- | --- | --- | --- | --- | --- | --- | --- | --- |
|  |  | |  |  |  |  | |  |  |
|  | **2013** | | | |  | **2015** | | | |
|  | OOPE on inpatient and outpatient care last year | OOPE on medicine last year | Total OOPE | % of OOPE on medicine of total OOPE |  | OOPE on inpatient and outpatient care last year | OOPE on medicine last year | Total OOPE | % of OOPE on medicine of total OOPE |
| Full sample | 235.9 | 95.3 | 331.2 | 50.2% |  | 242.8 | 94.7 | 337.5 | 52.8% |
| **Age group** |  |  |  |  |  |  |  |  |  |
| 50-59 years | 233.1 | 72.7 | 305.8 | 46.2% |  | 227.7 | 76.6 | 304.3 | 51.4% |
| 60-69 years | 253.6 | 92.1 | 345.7 | 48.5% |  | 261.5 | 89.7 | 351.2 | 50.2% |
| 70+ years | 224.5 | 117.8 | 342.3 | 55.0% |  | 241.5 | 115.5 | 357.0 | 56.3% |
| **Gender** |  |  |  |  |  |  |  |  |  |
| male | 226.6 | 86.1 | 312.7 | 49.3% |  | 238.7 | 86.7 | 325.4 | 51.7% |
| female | 243.8 | 103.1 | 347.0 | 50.9% |  | 246.4 | 101.6 | 348.0 | 53.8% |
| **Marital status** |  |  |  |  |  |  |  |  |  |
| other | 223.7 | 98.3 | 322.0 | 52.0% |  | 218.1 | 96.1 | 314.2 | 55.0% |
| married or in a civil partnership | 242.5 | 93.7 | 336.2 | 49.2% |  | 256.5 | 93.9 | 350.4 | 51.7% |
| **Educational attainment** | |  |  |  |  |  |  |  |  |
| less than upper secondary | 208.4 | 101.7 | 310.1 | 57.5% |  | 217.9 | 96.4 | 314.3 | 58.4% |
| upper secondary | 235.6 | 90.2 | 325.8 | 47.6% |  | 247.3 | 92.2 | 339.5 | 51.2% |
| above | 294.5 | 91.2 | 385.7 | 41.3% |  | 280.9 | 96.0 | 376.9 | 46.8% |
| **Household income** | |  |  |  |  |  |  |  |  |
| Q1 | 175.6 | 90.3 | 265.9 | 54.8% |  | 173.6 | 90.5 | 264.1 | 58.2% |
| Q2 | 208.7 | 97.4 | 306.1 | 52.7% |  | 205.3 | 98.0 | 303.2 | 56.3% |
| Q3 | 254.0 | 97.3 | 351.3 | 49.2% |  | 269.5 | 97.0 | 366.5 | 51.9% |
| Q4 | 309.5 | 96.5 | 406.0 | 44.4% |  | 323.0 | 93.6 | 416.7 | 45.8% |
| **Country** |  |  |  |  |  |  |  |  |  |
| Austria | 282.8 | 106.6 | 389.4 | 46.2% |  | 354.3 | 152.6 | 506.9 | 53.1% |
| Germany | 205.9 | 91.7 | 297.6 | 46.3% |  | 200.6 | 90.9 | 291.6 | 55.5% |
| Sweden | 280.5 | 110.6 | 391.1 | 31.8% |  | 284.5 | 110.3 | 394.7 | 31.9% |
| Spain | 187.0 | 63.2 | 250.2 | 72.7% |  | 175.3 | 58.8 | 234.0 | 68.2% |
| Italy | 347.3 | 138.0 | 485.3 | 46.5% |  | 381.6 | 127.3 | 508.9 | 44.2% |
| France | 160.7 | 48.0 | 208.6 | 51.8% |  | 145.4 | 48.4 | 193.8 | 53.8% |
| Denmark | 246.0 | 159.9 | 405.9 | 43.3% |  | 242.3 | 167.2 | 409.5 | 43.5% |
| Switzerland | 736.6 | 104.4 | 841.1 | 15.4% |  | 965.4 | 140.5 | 1,105.9 | 16.5% |
| Belgium | 243.5 | 232.7 | 476.2 | 48.4% |  | 198.4 | 242.3 | 440.8 | 53.0% |
| Czech Republic | 44.8 | 80.8 | 125.6 | 66.9% |  | 38.7 | 67.7 | 106.4 | 70.5% |
| Luxembourg | 382.0 | 179.9 | 561.9 | 41.1% |  | 377.3 | 207.8 | 585.1 | 39.4% |
| Slovenia | 83.4 | 31.4 | 114.9 | 53.3% |  | 81.2 | 40.5 | 121.7 | 68.0% |
| Estonia | 65.3 | 168.7 | 234.0 | 74.9% |  | 96.5 | 180.4 | 276.8 | 71.1% |
| Note: Descriptive statistics were calculated using the survey weights provided.  OOPE: out of pocket expenditures (in Euros); sample mean on OOPE was estimated among those who occurred relevant cost. | | | | | | | | | |

| **Table S5 Association between multimorbidity with OOPE on medicine among people ages 50 and older in 13 European countries between 2013-2015** | | | | | | |
| --- | --- | --- | --- | --- | --- | --- |
|  | **First part Logit** | | **Second part GLM** | | **Overall** | |
|  | Coefficient | 95% CI | Coefficient | 95% CI | Margins | 95% CI |
| Number of CDs (ref:0) |  |  |  |  |  |  |
| 1 | 0·78*** | (0·71 - 0·86) | 0·29*** | (0·23 - 0·35) | 42·71*** | (38·00 - 47·41) |
| 2 | 1·10*** | (1·02 - 1·19) | 0·48*** | (0·42 - 0·55) | 74·08*** | (68·17 - 79·99) |
| 3 | 1·29*** | (1·20 - 1·39) | 0·62*** | (0·56 - 0·68) | 99·31*** | (92·74 - 105·89) |
| 4+ | 1·44*** | (1·35 - 1·54) | 0·83*** | (0·77 - 0·88) | 140·73*** | (133·39 - 148·06) |
| Age groups (ref: 50-59 years) | | | | | | |
| 60-69 | -0·05 | (-0·12 - 0·03) | 0·05** | (0·01 - 0·10) | 4·96* | (-0·57 - 10·48) |
| 70+ | -0·09** | (-0·16 - -0·01) | 0·16*** | (0·12 - 0·21) | 16·99*** | (11·45 - 22·53) |
| Gender (ref: male) |  |  |  |  |  |  |
| Female | 0·24*** | (0·18 - 0·29) | 0·05*** | (0·02 - 0·09) | 12·76*** | (8·20 - 17·31) |
| Marital status (ref: other) | | | | | | |
| Married | 0·06* | (-0·00 - 0·12) | 0·07*** | (0·03 - 0·10) | 9·46*** | (4·78 - 14·13) |
| Educational attainment (ref: less than secondary school) | | | | | | |
| Upper secondary | 0·26*** | (0·18 - 0·34) | 0·03 | (-0·02 - 0·07) | 10·30*** | (4·61 - 15·98) |
| Tertiary | 0·28*** | (0·20 - 0·37) | 0·11*** | (0·05 - 0·16) | 21·03*** | (13·82 - 28·25) |
| Socio-economics position (ref: Q1 poorest) | | | | | | |
| Q2 | 0·25*** | (0·18 - 0·32) | -0·04* | (-0·08 - 0·00) | 2·41 | (-2·67 - 7·50) |
| Q3 | 0·36*** | (0·28 - 0·43) | -0·03 | (-0·07 - 0·02) | 6·81** | (1·07 - 12·55) |
| Q4 | 0·25*** | (0·17 - 0·33) | 0·04* | (-0·00 - 0·09) | 12·71*** | (6·50 - 18·92) |
| Country (ref: Austria) |  |  |  |  |  |  |
| Germany | 0·52*** | (0·43 - 0·61) | -0·61*** | (-0·67 - -0·56) | -50·63*** | (-57·78 - -43·47) |
| Sweden | 1·13*** | (1·02 - 1·24) | -0·43*** | (-0·47 - -0·38) | -18·77*** | (-26·12 - -11·41) |
| Spain | 0·29*** | (0·16 - 0·41) | -0·84*** | (-0·91 - -0·76) | -72·31*** | (-80·51 - -64·11) |
| Italy | 0·24*** | (0·14 - 0·33) | -0·04 | (-0·10 - 0·01) | 4·92 | (-3·84 - 13·67) |
| France | -0·42*** | (-0·51 - -0·33) | -0·84*** | (-0·90 - -0·77) | -87·24*** | (-94·38 - -80·09) |
| Denmark | 0·93*** | (0·84 - 1·03) | -0·09*** | (-0·14 - -0·03) | 23·37*** | (14·57 - 32·17) |
| Switzerland | -0·46*** | (-0·55 - -0·36) | 0·27*** | (0·21 - 0·34) | 12·92** | (1·85 - 23·99) |
| Belgium | 1·33*** | (1·23 - 1·44) | 0·19*** | (0·14 - 0·24) | 89·24*** | (79·06 - 99·41) |
| Czech Republic | 1·29*** | (1·15 - 1·44) | -0·97*** | (-1·03 - -0·92) | -67·14*** | (-74·32 - -59·96) |
| Luxembourg | 0·51*** | (0·38 - 0·64) | 0·13*** | (0·06 - 0·21) | 45·55*** | (31·29 - 59·81) |
| Slovenia | -0·69*** | (-0·79 - -0·60) | -0·97*** | (-1·06 - -0·89) | -98·86*** | (-105·98 - -91·73) |
| Estonia | 1·46*** | (1·36 - 1·55) | -0·17*** | (-0·22 - -0·13) | 22·88*** | (15·39 - 30·38) |
| Year |  |  |  |  |  |  |
| 2015 | 0·05** | (0·01 - 0·10) | 0·00 | (-0·03 - 0·03) | 1·52 | (-2·27 - 5·32) |
| Note: Estimates obtained from two-part model that the first part is modeled through a logit model to estimate the likelihood of incurring OOPE on medicine, and second part using a generalized linear model with gamma distribution and log link function to model the amount of OOPE on medicine if occurred.  GLM: generalised linear model; CD: chronic diseases; CI: confidence interval.  Standard errors were clustered at the individual level to control for serial correlation. Confidence interval in parentheses. *** statistical significance at the 1% level; ** statistical significance at the 5% level; *, statistical significance at the 10% level margins shows combined marginal effects from both parts of the two-part model. | | | | | | |

| **Table S6 Association between complex multimorbidity with OOPE on medicine among people ages 50 and older in 13 European countries between 2013-2015** | | | | | | |
| --- | --- | --- | --- | --- | --- | --- |
|  | **First part Logit** | | **Second part GLM** | | **Overall** | |
|  | Coefficient | 95% CI | Coefficient | 95% CI | Margins | 95% CI |
| Number of CDs from different body systems (ref:0) | | | | | | |
| 1 | 0·81*** | (0·74 - 0·88) | 0·31*** | (0·26 - 0·37) | 45·90*** | (41·20 - 50·61) |
| 2 | 1·13*** | (1·05 - 1·21) | 0·52*** | (0·46 - 0·58) | 79·43*** | (73·70 - 85·16) |
| 3 | 1·32*** | (1·23 - 1·42) | 0·66*** | (0·60 - 0·72) | 107·07*** | (100·56 - 113·59) |
| 4+ | 1·48*** | (1·37 - 1·59) | 0·86*** | (0·80 - 0·92) | 149·74*** | (140·78 - 158·70) |
| Age groups (ref: 50-59 years) | | | | | | |
| 60-69 | -0·04 | (-0·12 - 0·03) | 0·06** | (0·01 - 0·10) | 5·18* | (-0·35 - 10·71) |
| 70+ | -0·08** | (-0·16 - -0·01) | 0·16*** | (0·12 - 0·21) | 17·35*** | (11·82 - 22·88) |
| Gender (ref: male) |  |  |  |  |  |  |
| Female | 0·23*** | (0·18 - 0·29) | 0·05*** | (0·01 - 0·09) | 12·32*** | (7·76 - 16·88) |
| Marital status (ref: other) | | | | | | |
| Married | 0·06* | (-0·00 - 0·12) | 0·07*** | (0·03 - 0·10) | 9·51*** | (4·83 - 14·18) |
| Educational attainment (ref: less than secondary school) | | | | | | |
| Upper secondary | 0·26*** | (0·18 - 0·33) | 0·03 | (-0·02 - 0·07) | 10·04*** | (4·36 - 15·73) |
| Tertiary | 0·28*** | (0·19 - 0·37) | 0·10*** | (0·05 - 0·16) | 20·63*** | (13·43 - 27·82) |
| Socio-economics position (ref: Q1 poorest) | | | | | | |
| Q2 | 0·25*** | (0·18 - 0·32) | -0·04** | (-0·08 - -0·00) | 2·05 | (-3·05 - 7·16) |
| Q3 | 0·35*** | (0·28 - 0·43) | -0·03 | (-0·07 - 0·02) | 6·64** | (0·87 - 12·40) |
| Q4 | 0·25*** | (0·17 - 0·33) | 0·04 | (-0·01 - 0·09) | 11·91*** | (5·69 - 18·13) |
| Country (ref: Austria) |  |  |  |  |  |  |
| Germany | 0·52*** | (0·43 - 0·61) | -0·62*** | (-0·67 - -0·57) | -51·32*** | (-58·52 - -44·12) |
| Sweden | 1·12*** | (1·02 - 1·23) | -0·43*** | (-0·48 - -0·38) | -19·85*** | (-27·23 - -12·46) |
| Spain | 0·29*** | (0·16 - 0·41) | -0·84*** | (-0·91 - -0·76) | -72·82*** | (-81·08 - -64·57) |
| Italy | 0·23*** | (0·13 - 0·32) | -0·05* | (-0·10 - 0·01) | 3·60 | (-5·17 - 12·38) |
| France | -0·43*** | (-0·52 - -0·34) | -0·85*** | (-0·92 - -0·78) | -88·65*** | (-95·82 - -81·48) |
| Denmark | 0·93*** | (0·83 - 1·02) | -0·09*** | (-0·14 - -0·04) | 23·21*** | (14·32 - 32·10) |
| Switzerland | -0·47*** | (-0·56 - -0·37) | 0·26*** | (0·20 - 0·33) | 11·02* | (-0·01 - 22·05) |
| Belgium | 1·33*** | (1·22 - 1·43) | 0·19*** | (0·13 - 0·24) | 88·30*** | (78·11 - 98·50) |
| Czech Republic | 1·29*** | (1·15 - 1·43) | -0·97*** | (-1·03 - -0·91) | -67·46*** | (-74·74 - -60·17) |
| Luxembourg | 0·50*** | (0·37 - 0·63) | 0·13*** | (0·05 - 0·20) | 43·90*** | (29·67 - 58·14) |
| Slovenia | -0·69*** | (-0·79 - -0·60) | -0·98*** | (-1·06 - -0·90) | -99·84*** | (-106·98 - -92·70) |
| Estonia | 1·46*** | (1·36 - 1·55) | -0·17*** | (-0·21 - -0·12) | 23·90*** | (16·32 - 31·47) |
| Year |  |  |  |  |  |  |
| 2015 | 0·05** | (0·01 - 0·10) | 0 | (-0·03 - 0·03) | 1·58 | (-2·21 - 5·37) |
| Note: Estimates obtained from two-part model that the first part is modeled through a logit model to estimate the likelihood of incurring OOPE on medicine, and second part using a generalized linear model with gamma distribution and log link function to model the amount of OOPE on medicine if occurred.  GLM: generalised linear model; CD: chronic diseases; CI: confidence interval.  Standard errors were clustered at the individual level to control for serial correlation. Confidence interval in parentheses. *** statistical significance at the 1% level; ** statistical significance at the 5% level; *, statistical significance at the 10% level margins shows combined marginal effects from both parts of the two-part model. | | | | | | |

| **Table S7 Association between multimorbidity with OOPE on medicine among people ages 50 and older in 13 European countries between 2013-2015, using Cragg’s hurdle model** | | | | | | |
| --- | --- | --- | --- | --- | --- | --- |
|  | **Selection** | | **Outcome** | | **Overall** | |
|  | Coefficient | 95% CI | Coefficient | 95% CI | Margins | 95% CI |
| Number of CDs | 0·17*** | (0·17 - 0·17) | 0·18*** | (0·18 - 0·18) | 25·93*** | (25·46 - 26·40) |
| Age groups (ref: 50-59 years) | | | | | | |
| 60-69 | 0·01 | (-0·01 - 0·02) | 0·06*** | (0·05 - 0·08) | 6·16*** | (4·47 - 7·85) |
| 70+ | -0·02* | (-0·03 - 0·00) | 0·18*** | (0·17 - 0·20) | 17·58*** | (15·76 - 19·40) |
| Gender (ref: male) |  |  |  |  |  |  |
| Female | 0·14*** | (0·12 - 0·15) | 0·06*** | (0·05 - 0·08) | 12·70*** | (11·29 - 14·12) |
| Marital status (ref: other) | | | | | | |
| Married | 0·03*** | (0·02 - 0·05) | 0·06*** | (0·05 - 0·08) | 7·97*** | (6·48 - 9·46) |
| Educational attainment (ref: less than secondary school) | | | | | | |
| Upper secondary | 0·15*** | (0·13 - 0·17) | 0·04*** | (0·02 - 0·05) | 10·43*** | (8·59 - 12·28) |
| Tertiary | 0·16*** | (0·14 - 0·18) | 0·11*** | (0·09 - 0·13) | 18·47*** | (16·15 - 20·79) |
| Socio-economics position (ref: Q1 poorest) | | | | | | |
| Q2 | 0·15*** | (0·13 - 0·17) | -0·03*** | (-0·05 - -0·01) | 4·42*** | (2·53 - 6·32) |
| Q3 | 0·21*** | (0·20 - 0·23) | -0·02** | (-0·04 - -0·00) | 8·17*** | (6·22 - 10·12) |
| Q4 | 0·15*** | (0·13 - 0·17) | 0·04*** | (0·02 - 0·05) | 10·96*** | (8·86 - 13·06) |
| Country (ref: Austria) |  |  |  |  |  |  |
| Germany | 0·33*** | (0·30 - 0·35) | -0·65*** | (-0·67 - -0·63) | -57·82*** | (-60·81 - -54·83) |
| Sweden | 0·67*** | (0·65 - 0·70) | -0·26*** | (-0·28 - -0·24) | 2·55 | (-0·70 - 5·80) |
| Spain | 0·17*** | (0·14 - 0·20) | -0·94*** | (-0·97 - -0·92) | -85·37*** | (-88·71 - -82·03) |
| Italy | 0·14*** | (0·12 - 0·16) | -0·00 | (-0·02 - 0·02) | 11·34*** | (7·53 - 15·14) |
| France | -0·24*** | (-0·26 - -0·22) | -1·03*** | (-1·05 - -1·01) | -102·96*** | (-105·91 - -100·01) |
| Denmark | 0·57*** | (0·55 - 0·59) | -0·22*** | (-0·25 - -0·20) | 3·79** | (0·29 - 7·29) |
| Switzerland | -0·29*** | (-0·31 - -0·26) | 0·18*** | (0·15 - 0·21) | -0·54 | (-4·92 - 3·85) |
| Belgium | 0·80*** | (0·77 - 0·82) | 0·06*** | (0·04 - 0·08) | 68·28*** | (64·15 - 72·41) |
| Czech Republic | 0·78*** | (0·74 - 0·81) | -0·97*** | (-0·99 - -0·95) | -71·85*** | (-75·02 - -68·67) |
| Luxembourg | 0·32*** | (0·29 - 0·35) | -0·15*** | (-0·18 - -0·12) | 0·81 | (-4·42 - 6·04) |
| Slovenia | -0·41*** | (-0·43 - -0·38) | -1·14*** | (-1·16 - -1·11) | -112·26*** | (-115·21 - -109·32) |
| Estonia | 0·87*** | (0·85 - 0·89) | -0·18*** | (-0·20 - -0·17) | 23·42*** | (20·13 - 26·72) |
| Year |  |  |  |  |  |  |
| 2015 | 0·03*** | (0·02 - 0·05) | 0·04*** | (0·03 - 0·05) | 5·81*** | (4·43 - 7·20) |
| Note: Estimates obtained from Cragg’s Hurdle model. CD: chronic diseases; CI: confidence interval.  Standard errors were clustered at the individual level to control for serial correlation. Confidence interval in parentheses. *** statistical significance at the 1% level; ** statistical significance at the 5% level; *, statistical significance at the 10% level | | | | | | |

| **Table S8 Association between complex multimorbidity with OOPE on medicine among people ages 50 and older in 13 European countries between 2013-2015, using Cragg’s hurdle model** | | | | | | |
| --- | --- | --- | --- | --- | --- | --- |
|  | **First part Logit** | | **Second part GLM** | | **Overall** | |
|  | Coefficient | 95% CI | Coefficient | 95% CI | Margins | 95% CI |
| Number of CD from different body systems | 0·21*** | (0·21 - 0·22) | 0·21*** | (0·21 - 0·22) | 31·38*** | (30·81 - 31·95) |
| Age groups (ref: 50-59 years) | | | | | | |
| 60-69 | 0·00 | (-0·02 - 0·02) | 0·06*** | (0·04 - 0·08) | 5·57*** | (3·87 - 7·26) |
| 70+ | -0·03*** | (-0·05 - -0·01) | 0·18*** | (0·16 - 0·20) | 16·73*** | (14·90 - 18·56) |
| Gender (ref: male) |  |  |  |  |  |  |
| Female | 0·13*** | (0·12 - 0·15) | 0·06*** | (0·05 - 0·07) | 11·98*** | (10·57 - 13·40) |
| Marital status (ref: other) | | | | | | |
| Married | 0·03*** | (0·02 - 0·05) | 0·06*** | (0·05 - 0·08) | 7·85*** | (6·36 - 9·34) |
| Educational attainment (ref: less than secondary school) | | | | | | |
| Upper secondary | 0·15*** | (0·13 - 0·17) | 0·04*** | (0·02 - 0·05) | 10·33*** | (8·49 - 12·17) |
| Tertiary | 0·16*** | (0·14 - 0·18) | 0·11*** | (0·09 - 0·12) | 18·41*** | (16·10 - 20·73) |
| Socio-economics position (ref: Q1 poorest) | | | | | | |
| Q2 | 0·15*** | (0·13 - 0·17) | -0·03*** | (-0·05 - -0·01) | 4·26*** | (2·36 - 6·17) |
| Q3 | 0·21*** | (0·19 - 0·23) | -0·02*** | (-0·04 - -0·01) | 7·70*** | (5·75 - 9·66) |
| Q4 | 0·15*** | (0·13 - 0·17) | 0·03*** | (0·01 - 0·05) | 9·91*** | (7·81 - 12·01) |
| Country (ref: Austria) |  |  |  |  |  |  |
| Germany | 0·32*** | (0·30 - 0·34) | -0·66*** | (-0·67 - -0·64) | -58·60*** | (-61·60 - -55·59) |
| Sweden | 0·67*** | (0·64 - 0·69) | -0·27*** | (-0·29 - -0·25) | 1·23 | (-2·03 - 4·49) |
| Spain | 0·17*** | (0·14 - 0·20) | -0·94*** | (-0·97 - -0·91) | -85·65*** | (-89·01 - -82·28) |
| Italy | 0·13*** | (0·11 - 0·16) | -0·01 | (-0·03 - 0·01) | 9·58*** | (5·77 - 13·40) |
| France | -0·25*** | (-0·27 - -0·23) | -1·04*** | (-1·07 - -1·02) | -104·56*** | (-107·52 - -101·60) |
| Denmark | 0·56*** | (0·54 - 0·59) | -0·24*** | (-0·26 - -0·21) | 1·69 | (-1·82 - 5·20) |
| Switzerland | -0·29*** | (-0·31 - -0·27) | 0·17*** | (0·15 - 0·20) | -2·29 | (-6·67 - 2·10) |
| Belgium | 0·79*** | (0·77 - 0·82) | 0·06*** | (0·03 - 0·08) | 66·08*** | (61·94 - 70·23) |
| Czech Republic | 0·77*** | (0·74 - 0·81) | -0·97*** | (-1·00 - -0·95) | -72·48*** | (-75·69 - -69·27) |
| Luxembourg | 0·30*** | (0·27 - 0·33) | -0·16*** | (-0·19 - -0·13) | -1·40 | (-6·65 - 3·84) |
| Slovenia | -0·41*** | (-0·44 - -0·39) | -1·14*** | (-1·17 - -1·11) | -113·19*** | (-116·15 - -110·23) |
| Estonia | 0·87*** | (0·85 - 0·89) | -0·18*** | (-0·20 - -0·16) | 23·56*** | (20·24 - 26·89) |
| Year |  |  |  |  |  |  |
| 2015 | 0·03*** | (0·02 - 0·05) | 0·05*** | (0·03 - 0·06) | 6·15*** | (4·77 - 7·54) |
| Note: Estimates obtained from Cragg’s Hurdle model. CD: Long term condition; CI: confidence interval.  Standard errors were clustered at the individual level to control for serial correlation. Confidence interval in parentheses. *** statistical significance at the 1% level; ** statistical significance at the 5% level; *, statistical significance at the 10% level | | | | | | |
